# Rare Mutations of Peroxisome Proliferator-Activated Receptor Gamma: Frequencies and Relationship with Insulin Resistance and Diabetes Risk in the Mixed Ancestry Population from South Africa

**DOI:** 10.1155/2014/187985

**Published:** 2014-08-14

**Authors:** Z. Vergotine, A. P. Kengne, R. T. Erasmus, Y. Y. Yako, T. E. Matsha

**Affiliations:** ^1^Biomedical Sciences, Faculty of Health and Wellness Sciences, Cape Peninsula University of Technology, P.O. Box 1906, Bellville, Cape Town 7530, South Africa; ^2^Division of Chemical Pathology, Stellenbosch University, Cape Town 7505, South Africa; ^3^Non-Communicable Diseases Research Unit, South African Medical Research Council and University of Cape Town, Cape Town 7505, South Africa

## Abstract

*Background*. Genetic variants in the nuclear transcription receptor, PPARG, are associated with cardiometabolic traits, but reports remain conflicting. We determined the frequency and the clinical relevance of PPARG SNPs in an African mixed ancestry population. *Methods*. In a cross-sectional study, 820 participants were genotyped for rs1800571, rs72551362, rs72551363, rs72551364, and rs3856806, using allele-specific TaqMan technology. The homeostatic model assessment of insulin (HOMA-IR), *β*-cells function (HOMA-B%), fasting insulin resistance index (FIRI), and the quantitative insulin-sensitivity check index (QUICKI) were calculated. *Results*. No sequence variants were found except for the rs3856806. The frequency of the PPARG-His447His variant was 23.8% in the overall population group, with no difference by diabetes status (*P* = 0.215). The His447His allele T was associated with none of the markers of insulin resistance overall and by diabetes status. In models adjusted for 2-hour insulin, the T allele was associated with lower prevalent diabetes risk (odds ratio 0.56 (95% CI 0.31–0.95)). *Conclusion*. Our study confirms the almost zero occurrences of known rare PPARG SNPs and has shown for the first time in an African population that one of the common SNPs, His447His, may be protective against type 2 diabetes.

## 1. Introduction

Peroxisome proliferator-activated receptor gamma (PPARG) is one of the three PPARs isotypes; the other two are PPAR*α* and PPAR*β*/*δ*. The PPARG is mainly expressed in adipose tissue whilst PPAR*α* is expressed mostly in brown adipose tissue and liver, and PPAR*β*/*δ*, which is found in many tissues, is mostly expressed in gut, kidney, and heart [1, 2]. Expression of PPARs occurs when heterodimers are formed with the retinoid X receptor (RXR) and binds to specific PPAR responsive elements of DNA to promote transcription. The PPAR: RXR complex is activated via ligand binding to PPARs resulting in the release of corepressors bound to the receptor and recruitment of coactivators to initiate transcription of target genes [3–5]. For example, antidiabetic drugs of the thiazolidinedione family are believed to target the transcription factor PPARG to improve insulin sensitivity in type 2 diabetes (T2D) and induce glucose transporter 4 mRNA expression in fat and muscle [6–8].

Naturally occurring PPARG polymorphisms have been described in several studies. The two common variants of the PPARG gene, Pro12Ala and His447His, have been associated with metabolic states of obesity, insulin resistance, type 2 diabetes, and metabolic syndrome [9]. The His447His polymorphism (also referred to as C161T, C1431T, or CAC478CAT, His449His) is a silent mutation at exon 6 and is considered a better predictor of fasting insulin levels and insulin resistance than Pro12Ala [10]. In our previous analysis, the presence of* PPARG* Pro12 conferred a 64% higher risk of prevalent type 2 diabetes, but we failed to demonstrate an association with markers of insulin resistance [11]. In the present study, we therefore investigated the association between cardiometabolic traits, His447His, and other PPARG polymorphisms.

## 2. Materials and Methods

### 2.1. Baseline Evaluations

This investigation is based on the Bellville South cohort from Cape Town, South Africa, which has received study approval from the Research Ethics Committees of the University of Stellenbosch (HREC reference number N09/05/146) and Cape Peninsula University of Technology Faculty of Health and Wellness Sciences Ethics Committee (reference numbers CPUT/HW-REC 2008/002 and CPUT/HW-REC 2010). The study was conducted according to the code of ethics of the World Medical Association (Declaration of Helsinki). A detailed description of the research setting has been previously described [12, 13]. Briefly, Bellville South is predominantly a mixed ancestry township located in the northern suburb of Cape Town in the Tygerberg subdistrict. The mixed ancestry, commonly referred to as coloured, is a South African population group with 32–43% Khoisan, 20–36% Bantu-speaking African, 21–28% European, and 9–11% Asian ancestry [14]. All participants received a standardized interview and physical examination during which blood pressure was measured according to the World Health Organisation (WHO) guidelines [15] using a semiautomated digital blood pressure monitor (Rossmax PA, USA) on the right arm in a sitting position. Anthropometric measurements were performed three times and their average was used for analysis: weight (kg), height (cm), waist (cm), and hip (cm) circumferences. Participants with no history of doctor-diagnosed diabetes mellitus underwent a 75 g oral glucose tolerance test (OGTT) as recommended by the WHO [16]. Further, the following biochemical parameters were determined on the Cobas 6000 Clinical Chemistry instrument (Roche Diagnostics, Germany): fasting plasma glucose, insulin, creatinine, total cholesterol (TC), high density lipoprotein cholesterol (HDL-c), triglycerides (TG), C-reactive protein (CRP), *γ*-glutamyltransferase (GGT), and glycated haemoglobin (HbA1c) certified by the National Glycohemoglobin Standardisation Programme (NGSP). Low density lipoprotein cholesterol (LDL-c) was calculated using Friedewald's formula [17].

### 2.2. SNP Genotyping

Genomic DNA was extracted from whole blood samples collected in an EDTA tube. Single nucleotide polymorphisms (SNPs) in the PPARG, Pro115Gln (rs1800571, G>T), Val290Met (rs72551362, G>A), Pheu388Leu (rs72551363, T>A), Arg397Cys (rs72551364, C>T), and His447His (rs3856806, C>T), were genotyped using high throughput real-time polymerase chain reaction (RT-PCR) in two independent laboratories on the ABI Prism 7900HT platform (Applied Biosystems, USA) and a Bio-Rad Optica (BioRad, USA) using TaqMan genotyping assay (Applied Biosystems, USA). Conventional polymerase chain reaction followed by direct DNA sequencing was performed for analytical validation of high throughput genotyping.

### 2.3. Definitions and Calculations

Body mass index (BMI) was calculated as weight per square meter (kg/m^2^) and waist-hip-ratio (WHR) as waist/hip circumferences (cm). Type 2 diabetes status was based on a history of doctor-diagnosis, a fasting plasma glucose ≥7.0 mmol/L, and/or a 2-hour post-OGTT plasma glucose ≥11.1 mmol/L. The homeostatic model assessment of insulin resistance (HOMA-IR) was calculated according to the formula HOMA-IR = fasting insulin concentration (mIU/L) × fasting plasma glucose (mmol/L)/22.5, while functional *β*-cells (HOMA-B%) were estimated using the formula 20 × fasting insulin (*μ*IU/mL)/fasting glucose (mmol/mL) − 3.5. The fasting insulin resistance index (FIRI) was calculated with the formula (fasting insulin (*μ*U/mL) × fasting glucose (mM))/25 and the quantitative insulin-sensitivity check index (QUICKI) was calculated as 1/log fasting insulin (*μ*U/mL) × log (fasting glucose (mg/dL)). Glomerular filtration rate (GFR) was estimated by the 4-variable modification of diet in renal disease (MDRD) equation [18, 19] applicable to standardised serum creatinine values.

### 2.4. Statistical Analysis

Of the 946 participants who took part in the survey, 941 consented for genetic studies. Among the latter, 121 were excluded for missing data on the genetic variables. Therefore, 820 had valid data for the current analyses. General characteristics of the study group are summarized as count and percentage for dichotomous traits, mean and standard deviation (SD), or median and 25th–75th percentiles for quantitative traits. Traits were log-transformed to approximate normality, where necessary, prior to analysis. SNPs were tested for departure from Hardy-Weinberg equilibrium (HWE) expectation via a chi-square goodness of fit test. Linear regression models were used for the analysis of quantitative traits and logistic regression models for dichotomous traits, always assuming additive models for the SNPs. Using linear and logistic models enabled us to adjust all analyses for known confounders as specified everywhere in the results. We investigated the additive allelic association of each SNP with each trait, overall and according to type 2 diabetes status, and tested for heterogeneity by adding the interaction term of type 2 diabetes and each SNP to a model that contained the main effects of type 2 diabetes and the relevant SNP. Results corresponding to *P* values below 5% are described as significant. We did not adjust for multiple testing. All analyses used the statistical package R (version 3.0.0 (2013-04-03), The R Foundation for Statistical Computing, Vienna, Austria).

## 3. Results

Clinical characteristics of participants overall and according to diabetes status are summarized in Table 1 indicating that 222 (27%) participants had type 2 diabetes. As expected, the distribution of the level of insulin resistance/sensitivity indicators was significantly different between the two groups all *P* < 0.0001, except for glucose/insulin ratio (*P* = 0.0304). Furthermore, compared with nondiabetic participants, those with diabetes were significantly older (58.9 versus 51.0 years, *P* < 0.0001) and had higher levels of adipometric variables (all *P* ≤ 0.014), systolic blood pressure (*P* < 0.0001), triglycerides (*P* < 0.0001), GGT, and CRP (both *P* < 0.0001), whilst eGFR (*P* = 0.029) and HDL cholesterol (*P* = 0.0002) were significantly lower.

No mutations were found for the following SNPs of PPARG: Pro115Gln, Val290Met, Pheu388Leu, and Arg397Cys. The PPARG, His447His, variant was in HWE (*P* > 0.05) and its genotype and allele distribution overall and by diabetes status are summarized in Table 2. The frequency of the PPARG (rs3856806), C/T genotype, was 23.8% in the overall population group and the genotype distributions did not differ significantly between the nondiabetic and diabetic groups. Overall, the T allele frequency was 14%; however, although not significant (*P* = 0.128), the T allele did occur more in the nondiabetic (14.8%) than in the diabetic (11.7%) participants.

In generalized linear regression analyses adjusted for age, sex and diabetes (Table 3), PPARG His447His allele T was associated with none of the markers of insulin resistance overall and by diabetes status. There was a borderline statistical interaction in the effect by diabetes status for the association with fasting glucose (interaction *P* = 0.092), HOMA-IR and FIRI indices (both *P* = 0.076). Furthermore, the effects always appeared to be sizable and negative (although nonsignificant) among the diabetic and almost null in nondiabetic participants.

In the logistic regression models (Table 4) PPARG allele T was associated with odds ratio of 0.76 (95% confidence interval 0.55–1.05) for prevalent diabetes. The effect estimates broadly remained within the same range after adjustment for age and sex, with or without further adjustment for various indices of insulin resistance/sensitivity. However, in model containing sex, age, and 2-hour insulin, PPARG allele T was significantly associated with a lower prevalent diabetes risk with an odds ratio of 0.56 (95% CI: 0.31–0.95).

## 4. Discussion

The present study reports five PPARG SNPs, rs1800571, rs72551362, rs72551363, rs72551364, and rs3856806. Only His447His (rs3856806) was found to be polymorphic and demonstrated an association with diabetes risk. The T allele frequencies were about 14% and, in an additive genetic model, we observed that the presence of the T allele significantly reduced the risk of diabetes by 44%. Furthermore, the T allele was associated with reduced fasting blood glucose levels and HOMA-IR and FIRI indices. However these associations were borderline and did not reach statistical significance.

The nuclear transcription receptor, PPARG, acts as a regulator of adipocyte differentiation, pancreatic beta cell function, and lipid and glucose metabolism [20–22]. Despite its biological plausibility, previously reported association studies with obesity [23–25], insulin resistance [10, 25], type 2 diabetes [11, 26], and metabolic syndrome [27] are conflicting. For instance, some studies have reported that carriers of the T allele of His447His (rs3856806) have increased obesity risk [23], poor lipid profile [28–30], and risk of metabolic syndrome [27] and coronary heart diseases (CHD) [9]. On the other hand, others have failed to demonstrate an association between PPARG SNPs including C161T with CHD susceptibility [31], whilst some have shown increased insulin sensitivity [10, 26], decreased CHD, body mass index, and diabetes risk [25, 26, 29, 32] in subjects harbouring the T allele. In our study, presence of the T allele was associated with reducing the risk of prevalent diabetes and levels of insulin resistance indices. Aspects that may contribute to the inconsistencies in these studies include variations in ethnic distribution of PPARG polymorphisms, the statistical power of the studies, environmental-gene and gene-gene factors, and different levels of adjustment for potential confounding factors across studies. For example, in our study, the T allele frequency of the His447His variant (14%) was similar to that reported in the Australian Caucasians, 16.3% [2], and the Tunisians, 18.3% [27], whilst in the Chinese a frequency of 29.2% has been reported [33]. Furthermore, two recent studies have shown that gene-gene interaction analysis is superior to single PPARG polymorphisms for the quantification of effects with cardiometabolic traits [33, 34]. For example, Luo et al. [33] investigated the association of 10 PPARG SNPs with obesity and found that only two SNPs, rs2016520 and rs10865170, were associated with lower obesity risk. However, in generalized multifactor dimensionality reduction analysis to assess the effect of interaction among the 10 SNPs, an additional SNP, rs9794, was identified and showed a potential gene-gene interaction with rs2016520 and rs10865170 [33].

We also genotyped some of the PPARG SNPs, rs1800571, rs72551362, rs72551363, and rs72551364, but no genetic variations were observed. The rs1800571, Pro115Gln, gain-of-function mutation has only been found in five German morbid obese subjects since its initial identification by Ristow et al. [35], where three of the four obese subjects with the genetic variant also had type 2 diabetes. In another German population study, another carrier of the mutant with severe insulin resistance was identified [36]. However, other studies involving German, Danish, and American populations failed to find this mutation [37–40]. Similarly, there has not been more than one individual in which either rs72551362, rs72551363, or rs72551364 has been found [41–43]. The rare nature of these mutations suggests that standard population-based type studies are likely not the suitable design to investigate them, considering the requirements for very large sample size. Rather, targeting specific segments of the population such as people with morbid obesity for screening may increase the likelihood of uncovering some with the mutation. It is also possible that mutations other than those reported so far could be present in other populations, indicating the need for genome-wide scanning studies in different setting to capture additional mutations.

Our study has some limitations and these include the investigation of only five SNPs and small sample size. However, the participants in this study were well characterised and we used both fasting and oral glucose tolerance test derived indices for the assessment of type 2 diabetes and insulin resistance. In conclusion, our study confirmed the almost zero occurrences of known rare PPARG SNPs and has shown that one of the common SNPs, His447His, may be protective against type 2 diabetes. Future larger studies with more SNPs involving populations from Africa need further exploration.

## Figures and Tables

**Table 1:** General characteristics of the overall population and by diabetic status.

Variable	No diabetes	Diabetes	*P*-value	Overall
Number	**598**	**222**		**820**
Gender, male *n* (%)	130 (21.7)	48 (21.6)	0.9474	178 (21.7)
Mean age, year (SD)	51.0 (15.1)	58.9 (13.4)	<0.0001	53.2 (15.1)
Mean systolic blood pressure, mmHg (SD)	122 (19)	130 (23)	<0.0001	124 (21)
Mean diastolic blood pressure, mmHg (SD)	75 (12)	77 (14)	0.0506	76 (13)
Hypertension, *n* (%)	314 (52.5)	133 (59.9)	0.05859	447 (54.5)
Mean body mass index, kg/m^2^ (SD)	29.2 (7.0)	31.8 (7.1)	<0.0001	29.9 (7.1)
Mean waist circumference, cm (SD)	95 (15)	102 (14)	<0.0001	97 (15)
Mean hip circumference, cm (SD)	109 (14)	112 (15)	0.014	109 (14)
Mean waist/hip ratio, (SD)	0.87 (0.10)	0.91 (0.10)	<0.0001	0.88 (0.09)
Mean HbA1c, % (SD)	5.7 (0.4)	7.8 (2.1)	<0.0001	6.3 (1.4)
Mean fasting blood glucose, mmol/L (SD)	5.1 (0.7)	9.8 (4.5)	<0.0001	6.4 (3.2)
Mean 2 h glucose, mmol/L (SD)	6.4 (1.6)	13.4 (5.3)	<0.0001	7.5 (3.6)
Mean eGFR, mL/min (SD)	76.2 (21.2)	72.0 (25.2)	0.029	75.0 (22.4)
Mean triglycerides, mmol/L (SD)	1.4 (0.9)	1.7 (0.9)	<0.0001	1.5 (0.9)
Mean HDL cholesterol, mmol/L (SD)	1.3 (0.4)	1.2 (0.3)	0.0002	1.3 (0.4)
Mean LDL cholesterol, mmol/L (SD)	3.6 (1.0)	3.7 (1.1)	0.179	3.6 (1.0)
Mean total cholesterol, mmol/L (SD)	5.5 (1.2)	5.7 (1.3)	0.068	5.6 (1.2)
Median GGT (25th–75th percentiles)	26 [17–39]	32 [23–49]	<0.0001	27 [19–42]
Median CRP (25th–75th percentiles)	3.3 [0.9–8.3]	5.4 [2.2–10.9]	<0.0001	4.0 [1.2–9.4]
Median insulin mmol/L (25th–75th percentiles)	7.2 [3.4–12.9]	10.2 [4.4–17.6]	0.0001	7.7 [3.5–14.1]
Median 2 h insulin mmol/L (25th–75th percentiles)	36.0 [19.3–65.7]	53.3 [22.1–117.0]	0.0015	37.3 [19.5–72.7]
Median glucose/insulin (25th–75th percentiles)	0.71 [0.40–1.45]	0.82 [0.48–2.05]	0.0304	0.72 [0.41–1.58]
Median HOMA-IR (25th–75th percentiles)	1.63 [0.69–3.00]	3.67 [1.71–7.29]	<0.0001	1.94 [0.86–3.82]
Median HOMA-B% (25th–75th percentiles)	92.8 [43.5–165.1]	42.1 [13.6–83.4]	<0.0001	74.2 [32.2–149.2]
Median QUICKI (25th–75th percentiles)	0.15 [0.14–0.18]	0.14 [0.12–0.15]	<0.0001	0.15 [0.14–0.17]
Median FIRI (25th–75th percentiles)	1.46 [0.62–2.70]	3.31 [1.54–6.56]	<0.0001	1.74 [0.77–3.44]
Median 1/HOMA-IR (25th–75th percentiles)	0.61 [0.33–1.44]	0.27 [0.14–0.58]	<0.0001	0.52 [0.26–1.16]

CRP: C-reactive protein; eGFR: estimated glomerular filtration rate; FIRI: fasting *insulin*-resistance index; GGT: *γ*-glutamyltransferase; HbA1c: glycated haemoglobin; HDL: high density lipoproteins; HOMA-B%: functional *β*-cells; HOMA-IR: homeostatic model assessment of insulin resistance; LDL: low density lipoproteins; QUICKI: the quantitative *insulin*-sensitivity check index; SD: standard deviation.

**Table 2:** Genotype distributions, minor allele frequencies, and unadjusted *P*-values for comparing genotype distribution according to diabetes status and additive allelic effects between diabetes groups.

	No diabetes	Diabetes	*P*-value	Overall
*N*	**598**	**222**		**820**
PPARG (rs3856806)				
C/C, *n* (%)	436 (72.9)	172 (77.5)	0.215	608 (74.1)
C/T, *n* (%)	147 (24.5)	48 (21.6)		195 (23.8)
T/T, *n* (%)	15 (2.5)	2 (0.9)		17 (2.1)
T, *n* (%)	177 (14.8)	52 (11.7)	0.128	229 (14.0)
HWE (*P*-value)	0.517	0.747		0.771

HWE: Hardy-Weinberg equilibrium (HWE *P*-values are from exact tests); PPARG: peroxisome proliferator-activated receptor gamma.

**Table 3:** Generalized linear regression models showing the effects of genes on markers of insulin resistance/sensitivity.

Allele	Phenotype	Overall		No diabetes		Diabetes		*P* interaction
		Effects size (95% CI)	*P*	Effects size (95% CI)	*P*	Effects size (95% CI)	*P*	
PPARG (rs3856806)T	Fasting glucose	−0.140 (−0.480 to 0.199)	0.417	0.0002 (−0.112 to 0.112)	0.996	−0.606 (−1.934 to 0.722)	0.372	0.092
	2 h glucose	−0.114 (−0.494 to 0.266)	0.557	−0.098 (−0.341 to 0.144)	0.426	−0.147 (−2.826 to 2.533)	0.915	0.847
	HbA1c	−0.018 (−0179 to 0.143)	0.826	0.034 (−0.031 to 0.099)	0.303	−0.211 (−0.829 to 0.407)	0.504	0.164
	Fasting insulin	−0.915 (−3.362 to 1.533)	0.464	0.022 (−1.420 to 1.463)	0.976	−4.516 (−13.448 to 4.416)	0.323	0.125
	2 h insulin	−1.716 (−11.818 to 8.386)	0.739	0.408 (−9.473 to 10.289)	0.936	−20.212 (−62.232 to 21.807)	0.348	0.190
	Glucose/insulin	−0.488 (−1.905 to 0.929)	0.500	−0.083 (−1.258 to 1.091)	0.890	−1.743 (−6.271 to 2.785)	0.451	0.334
	HOMA-IR	−0.437 (−1.481 to 0.608)	0.413	0.021 (−0.327 to 0.368)	0.907	−2.228 (−6.336 to 1.880)	0.289	0.076
	QUICKI	0.001 (−0.006 to 0.009)	0.738	0.0008 (−0.008 to 0.010)	0.851	0.004 (−0.009 to 0.016)	0.564	0.752
	FIRI	−0.393 (−1.333 to 0.547)	0.413	0.019 (−0.294 to 0.332)	0.907	−2.005 (−5.703 to 1.692)	0.289	0.076

Models are adjusted for age, sex, and diabetes. FIRI: fasting insulin resistance index; HOMA-IR: homeostatic model assessment of insulin resistance; PPARG: peroxisome proliferator-activated receptor gamma; QUICKI: the quantitative insulin-sensitivity check index.

**Table 4:** Odds ratio and 95% confidence intervals from logistic regression for the prediction of diabetes.

Allele	Covariates	OR (95% CI)	*P*
PPARG (rs3856806)T	None	0.76 (0.55 to 1.06)	0.112
	sex, age	0.74 (0.53 to 1.03)	0.082
	sex, age, insulin	0.75 (0.53 to 1.05)	0.098
	sex, age, 2 h insulin	0.56 (0.31 to 0.95)	0.042
	sex, age, HOMA-IR	0.77 (0.53 to 1.10)	0.157
	sex, age, QUICKI	0.75 (0.53 to 1.04)	0.094
	sex, age, FIRI	0.77 (0.53 to 1.10)	0.157
	sex, age, glucose/insulin	0.75 (0.53 to 1.04)	0.092

FIRI: fasting insulin resistance index; HOMA-IR: homeostatic model assessment of insulin resistance; PPARG: peroxisome proliferator-activated receptor; QUICKI: the quantitative insulin-sensitivity check index.

## References

[1] Desvergene B, Wahli W (1999). Peroxisome proliferator-activated receptors: nuclear control of metabolism. *Endocrine Reviews*.

[2] Wang XL, Oosterhof J, Duarte N (1999). Peroxisome proliferator-activated receptor *γ* C161→T polymorphism and coronary artery disease. *Cardiovascular Research*.

[3] Mukherjee R, Hoener PA, Jow L (2000). A selective peroxisome proliferator-activated receptor-*γ* (PPAR*γ*) modulator blocks adipocyte differentiation but stimulates glucose uptake in 3T3-L1 adipocytes. *Molecular Endocrinology*.

[4] Deeb SS, Fajas L, Nemoto M (1998). A Pro12Ala substitution in PPAR*γ*2 associated with decreased receptor activity, lower body mass index and improved insulin sensitivity. *Nature Genetics*.

[5] Pascual G, Fong AL, Ogawa S (2005). A SUMOylation-dependent pathway mediates transrepression of inflammatory response genes by PPAR-*γ*. *Nature*.

[6] Armoni M, Kritz N, Harel C (2003). Peroxisome proliferator-activated receptor-*γ* represses GLUT4 promoter activity in primary adipocytes, and rosiglitazone alleviates this effect. *Journal of Biological Chemistry*.

[7] Cariou B, Charbonnel B, Staels B (2012). Thiazolidinediones and PPAR*γ* agonists: time for a reassessment. *Trends in Endocrinology & Metabolism*.

[8] Yau H, Rivera K, Lomonaco R, Cusi K (2013). The future of thiazolidinedione therapy in the management of type 2 diabetes mellitus. *Current Diabetes Reports*.

[9] Ding S, Liu L, Zhuge Q (2012). The meta-analysis of the association of PPARG P12A, C161T polymorphism and coronary heart disease. *Wiener Klinische Wochenschrift*.

[10] Moffett SP, Feingold E, Barmada MM (2005). The C161→T polymorphism in peroxisome proliferator-activated receptor gamma, but not P12A, is associated with insulin resistance in Hispanic and non-Hispanic white women: evidence for another functional variant in peroxisome proliferator-activated receptor gamma. *Metabolism: Clinical and Experimental*.

[11] Vergotine Z, Yako YY, Kengne AP (2014). Proliferator-activated receptor gamma Pro12Ala interacts with the insulin receptor substrate 1 Gly972Arg and increase the risk of insulin resistance and diabetes in the mixed ancestry population from South Africa. *BMC Genetics*.

[12] Zemlin AE, Matsha TE, Hassan MS, Erasmus RT (2011). HbA1c of 6.5% to diagnose diabetes mellitus—does it work for us? The bellville South Africa study. *PLoS ONE*.

[13] Matsha TE, Soita DJ, Hassan MS (2013). Three-year’s changes in glucose tolerance status in the Bellville South cohort: rates and phenotypes associated with progression. *Diabetes Research and Clinical Practice*.

[14] de Wit E, Delport W, Rugamika CE (2010). Genome-wide analysis of the structure of the South African Coloured Population in the Western Cape. *Human Genetics*.

[15] Chalmers J, MacMahon S, Mancia G (1999). World Health Organization-international society of hypertension guidelines for the management of hypertension. Guidelines sub-committee of the World Health Organization. *Clinical and Experimental Hypertension*.

[16] Alberti KG, Zimmet PZ (1998). Definition, diagnosis and classification of diabetes mellitus and its complications. Part 1: diagnosis and classification of diabetes mellitus provisional report of a WHO consultation. *Diabetic Medicine*.

[17] Friedewald WT, Levy RI, Fredrickson DS (1972). Estimation of the concentration of low-density lipoprotein cholesterol in plasma, without use of the preparative ultracentrifuge. *Clinical Chemistry*.

[18] Levey AS, Bosch JP, Lewis JB, Greene T, Rogers N, Roth D (1999). A more accurate method to estimate glomerular filtration rate from serum creatinine: a new prediction equation. *Annals of Internal Medicine*.

[19] Levey AS, Coresh J, Greene T (2006). Chronic Kidney Disease Epidemiology Collaboration. Using standardized serum creatinine values in the modification of diet in renal disease study equation for estimating glomerular filtration rate. *Annals of Internal Medicine*.

[20] Spiegelman BM (1997). Peroxisome proliferator-activated receptor gamma: a key regulator of adipogenesis and systemic insulin sensitivity. *European Journal of Medical Research*.

[21] Rosen ED, Kulkarni RN, Sarraf P (2003). Targeted elimination of peroxisome proliferator-activated receptor *γ* in *β* cells leads to abnormalities in islet mass without compromising glucose homeostasis. *Molecular and Cellular Biology*.

[22] Hevener AL, He W, Barak Y (2003). Muscle-specific *Pparg* deletion causes insulin resistance. *Nature Medicine*.

[23] Valve R, Sivenius K, Miettinen R (1999). Two polymorphisms in the peroxisome proliferator-activated receptor-gamma gene are associated with severe overweight among obese women. *Journal of Clinical Endocrinology and Metabolism*.

[24] Meirhaeghe A, Fajas L, Helbecque N (1998). A genetic polymorphism of the peroxisome proliferator-activated receptor *γ* gene influences plasma leptin levels in obese humans. *Human Molecular Genetics*.

[25] Dongxia L, Qi H, Lisong L, Jincheng G (2008). Association of peroxisome proliferator-activated receptor*γ* gene Pro12Ala and C161T polymorphisms with metabolic syndrome. *Circulation Journal*.

[26] Tai ES, Corella D, Deurenberg-Yap M (2004). Differential effects of the C1431T and Pro12Ala PPAR*γ* gene variants on plasma lipids and diabetes risk in an Asian population. *Journal of Lipid Research*.

[27] Youssef SM, Mohamed N, Afef S (2014). Combined effects of the C161T and Pro12Ala PPAR*γ*2 gene variants with insulin resistance on metabolic syndrome: a case-control study of a central Tunisian population. *Journal of Molecular Neuroscience*.

[28] Gu SJ, Liu MM, Guo ZR (2013). Gene-gene interactions among PPAR*α*/*δ*/*γ* polymorphisms for hypertriglyceridemia in Chinese Han population. *Gene*.

[29] Yilmaz-Aydogan H, Kurnaz O, Kucukhuseyin O (2013). Different effects of PPARA, PPARG and ApoE SNPs on serum lipids in patients with coronary heart disease based on the presence of diabetes. *Gene*.

[30] Rhee EJ, Oh KW, Lee WY (2006). Effects of two common polymorphisms of peroxisome proliferator-activated receptor-gamma gene on metabolic syndrome. *Archives of Medical Research*.

[31] Xu W, Xu J, Sun B (2013). The effect of PPARG gene polymorphisms on the risk of coronary heart disease: a meta-analysis. *Molecular Biology Reports*.

[32] Liu Y, Yuan Z, Zhang J (2007). PPAR*γ* gene C161T substitution is associated with reduced risk of coronary artery disease and decreased proinflammatory cytokine expression. *The American Heart Journal*.

[33] Luo W, Guo Z, Wu M (2013). Association of peroxisome proliferator-activated receptor α/*δ*/*γ* with obesity, and gene-gene interaction, in the Chinese Han population. *Journal of Epidemiology*.

[34] Chan KH, Niu T, Ma Y (2013). Common genetic variants in peroxisome proliferator-activated receptor-*γ* (PPARG) and type 2 diabetes risk among Women's Health Initiative postmenopausal women. *The Journal of Clinical Endocrinology and Metabolism*.

[35] Ristow M, Müller-Wieland D, Pfeiffer A, Krone W, Kahn CR (1998). Obesity associated with a mutation in a genetic regulator of adipocyte differentiation. *The New England Journal of Medicine*.

[36] Blüher M, Paschke R (2003). Analysis of the relationship between PPAR-*γ* 2 gene variants and severe insulin resistance in obese patients with impaired glucose tolerance. *Experimental and Clinical Endocrinology & Diabetes*.

[37] Hamann A, Münzberg H, Buttron P (1999). Missense variants in the human peroxisome proliferator-activated receptor-gamma2 gene in lean and obese subjects. *European Journal of Endocrinology*.

[38] Evans D, Mann WA, De Heer J (2000). Variation in the gene for human peroxisome proliferator activated receptor *γ* (PPAR*γ*) does not play a major role in the development of morbid obesity. *International Journal of Obesity*.

[39] Ek J, Urhammer SA, Sørensen TIA, Andersen T, Auwerx J, Pedersen O (1999). Homozygosity of the Pro12Ala variant of the peroxisome proliferation-activated receptor-*γ*2 (PPAR-*γ*2): divergent modulating effects on body mass index in obese and lean Caucasian men. *Diabetologia*.

[40] Shuldiner AR, Nguyen W, Kao WHL (2000). Pro115Gln peroxisome proliferator-activated receptor-*γ* and obesity. *Diabetes Care*.

[41] Hegele RA, Cao H, Harris SB, Zinman B, Hanley AJG, Anderson CM (2000). Peroxisome proliferator-activated receptor-*γ*2 P12A and type 2 diabetes in Canadian Oji-Cree. *Journal of Clinical Endocrinology and Metabolism*.

[42] Barroso I, Gurnell M, Crowley VEF (1999). Dominant negative mutations in human PPAR*γ* associated with severe insulin resistance, diabetes mellitus and hypertension. *Nature*.

[43] Agarwal AK, Garg A (2002). A novel heterozygous mutation in peroxisome proliferator-activated receptor-*γ* gene in a patient with familial partial lipodystrophy. *The Journal of Clinical Endocrinology & Metabolism*.

